# Radiation-Induced Noncancer Risks in Interventional Cardiology: Optimisation of Procedures and Staff and Patient Dose Reduction

**DOI:** 10.1155/2013/976962

**Published:** 2013-08-20

**Authors:** Zhonghua Sun, Aini AbAziz, Ahmad Khairuddin Md Yusof

**Affiliations:** ^1^Discipline of Medical Imaging, Department of Imaging and Applied Physics, Curtin University, P.O. Box U1987, Perth, WA 6845, Australia; ^2^Department of Molecular Imaging and Nuclear Medicine, Universiti Kebangsaan Malaysia Medical Centre (UKMMC), Jalan Yaakob Latif, Cheras, 56000 Kuala Lumpur, Malaysia; ^3^Department of Cardiology, National Heart Institute, 50300 Kuala Lumpur, Malaysia

## Abstract

Concerns about ionizing radiation during interventional cardiology have been increased in recent years as a result of rapid growth in interventional procedure volumes and the high radiation doses associated with some procedures. Noncancer radiation risks to cardiologists and medical staff in terms of radiation-induced cataracts and skin injuries for patients appear clear potential consequences of interventional cardiology procedures, while radiation-induced potential risk of developing cardiovascular effects remains less clear. This paper provides an overview of the evidence-based reviews of concerns about noncancer risks of radiation exposure in interventional cardiology. Strategies commonly undertaken to reduce radiation doses to both medical staff and patients during interventional cardiology procedures are discussed; optimisation of interventional cardiology procedures is highlighted.

## 1. Introduction

Medical exposure from X-rays and nuclear medicine is the largest man-made source of radiation exposure, representing a mean effective dose of 1.0–3.0 mSv per head per year [1]. The worldwide population exposure from medical radiation has been shown to increase, and the use of procedures (both diagnostic and therapeutic) with a high radiation dose has been growing steadily [2–5]. Although interventional cardiac procedures account for 12% of all radiological examinations, they are responsible for delivering the highest radiation dose (up to 50% of the total collective effective dose) [6]. Thus, radiation exposure is a significant concern for interventional cardiologists and patients due to the increasing workloads and the complexity of procedures over the last decade [7, 8].

With fluoroscopy the patient is imaged in real time to guide minimally invasive procedures that form part of the diagnostic and interventional procedures, and this requires medical and technical staff to directly participate in the procedures. Patients undergoing interventional procedures in cardiology face radiation exposure in the order of a thousand or more times than that involved in conventional radiography [9]. Similarly, the interventional cardiologists encounter much more radiation than most other medical staff due to their working position being close to the X-ray beam and the patient (the source of scatter radiation). Therefore, interventional cardiologists must have a thorough knowledge of consequences of exposure to patients and personnel to ionizing radiation and methods of reducing staff and patient radiation exposure. Evaluation and followup of radiation doses received by the medical staff and patients should be considered an important part of quality assurance programmes for interventional cardiology procedures.

Radiation safety in the practice of interventional cardiology has been addressed by several professional bodies. In 2005, the American College of Cardiology (ACC) Foundation proposed the interventional cardiology guidelines which emphasized that physicians are responsible for minimizing the radiation injury hazard to their patients, professional staff, and themselves [10]. The UNSCEAR 2008 report states that fluoroscopic procedures represent the largest source of occupational exposure in medicine [11]. In 2009, the American Heart Association (AHA) Science Advisory recommended the reference doses of common cardiology examinations [12], and in 2010 the ACC committee also expressed the need for appropriate and optimal use of radiation techniques in cardiology [13].

This paper provides an overview of the radiation-induced noncancer risks during interventional cardiology procedures, with a focus on the radiation risks to interventional cardiologists and patients, as well as strategies commonly undertaken to reduce radiation exposure.

## 2. Radiation-Induced Effects and Risks to Interventional Cardiologists and Other Medical Staff

There are two main biological effects of ionizing radiation: stochastic effects, which include carcinogenic and genetic effects and deterministic effects (also called tissue reactions), which refer to an immediate and very predictable change to the tissue [14]. Stochastic effects are those for which the probability of an effect, rather than its severity, depends on the dose of radiation received [15]. Radiation-induced cancer and genetic effects are stochastic in nature and this has been well addressed in the literature [16]. Stochastic effects are believed not to have a dose threshold level because injury to a few cells, or even a single cell could theoretically result in the development of disease.

Deterministic effects occur when the dose exceeds a specific threshold. The severity of deterministic effects commonly increases with dose, as more cells are killed or damaged. Common examples of deterministic effects related to interventional cardiology are skin and hair changes [17], cataracts, and cardiovascular disease [18].

### 2.1. Radiation-Induced Cataracts

One of the most vital yet ill-defined effects associated with ionizing radiation exposure is the effect on the transparency of the eye lens, a pathology called radiation cataract. According to their anatomic location, cataract or presence of lens opacities can be classified into three main types: nuclear, cortical, and posterior subscapular [19]. Lens changes include small dots and vacuoles at early stage of cataract, and these lesions aggregate to form larger opacities at late stage of disease development. Although the sensitivity of the lens of the eye to high doses of ionizing radiation is well known, there exist uncertainties about the relation between radiation dose and cataracts. The National Council on Radiation Protection (NCRP) and the International Commission on Radiological Protection (ICRP) proposed guidelines on the view that cataractogenesis is a deterministic effect and requires a threshold radiation dose (currently 2 Gy) [14, 20]. However, radiation-induced cataracts are reported in populations exposed to much lower doses than the current standards, and this strongly suggests a stochastic hypothesis [21–23].

Occupational exposure to ionizing radiation and lens opacities has been reported for medical personnel, such as radiology technicians [24]. Earlier studies have demonstrated a significant increase in eye lens opacities among interventional cardiologists and medical staff in cardiac catheterization laboratories [25, 26]. Later reports based on experiences from different countries indicated that risk of lens opacities among interventional cardiologists was at least twice that of unexposed groups [27, 28].

In April 2011, the ICRP revised its lifetime eye dose threshold for cataract induction downwards from 2000 mSv to 500 mSv and the occupational annual dose limit from 150 mSv to 20 mSv in a year, averaged over defined periods of 5 years, with no single year exceeding 50 mSv [14, 29]. This recommendation is having an immediate impact on the new International Basic Safety Standard issued by the International Atomic Energy Agency (IAEA) and the upcoming Directive of the European Commission [30, 31]. The impact of these new recommendations on the practice of international cardiology is considered significant, since interventional cardiologists are potentially at risk of developing radiation-induced cataracts, depending on their level of exposure and the number and complexity of invasive cardiac procedures [32, 33].

Some epidemiological studies have been published on the risk of cataracts in interventional cardiology. Junk et al. in their first report consisting of 59 volunteer participants (radiologists and cardiologists) observed that a high frequency of 37.3% participants having small paracentral dot-like opacities in the posterior subcapsular regions of the lens, consistent with early signs of radiation damage, and 8% had diagnosis of cataracts [34]. This has been confirmed by a recent report examining 54 cardiologists and 69 nurses and technicians, with lens change found in 50% of interventional cardiologists and 41% of nurses and technicians compared with findings of similar lens changes in <10% of controls [35].

The Occupational Cataracts and Lens Opacities in Interventional Cardiology (O'CLOC) study represents a large scale epidemiological study with the aim of testing the existence of an increased risk of radiation-induced cataracts among interventional cardiologists compared with a control group of cardiologists not exposed to X-rays [33, 36]. Unlike these earlier studies, the O'CLOC study included 106 interventional cardiologists (including coronary interventional cardiologists and electrophysiologists) and 99 unexposed nonmedical workers. The study showed a significant excess risk of cataract for interventional cardiologists: 18% of posterior subcapsular lens opacities among interventional cardiologists were observed, while only 5% among control group were observed (*P* < 0.05). Regarding cumulative eye lens dose, results were consistent with this excess risk. Overall, 29% of the interventional cardiologists and 20% of the electrophysiologists had a cumulative dose exceeding 500 mSv. These findings indicate that according to the revised ICRP lifetime eye dose threshold of 500 mSv, >25% of these cardiologists may already at risk of developing early radiation-induced cataracts. Furthermore, electrophysiologists may have had higher annual doses than cardiologists in recent years due to less use of eye protection equipment. These findings reinforce those reports based on small sample sizes and highlight the importance of increasing cardiologists' awareness of the regular use of radiation protection devices and the necessity of optimizing procedures for dose reduction.

The Optimization of RAdiation protection for MEDical staff (ORAMED) project is funded by EU-EURATOM within the 7° Framework Programme with the aim of studying the dose received by operators in some selected practices of diagnostic and interventional radiology and nuclear medicine procedures [37, 38]. The WP1 (working package) of the project is devoted to studying the eye lens and extremity doses in interventional radiology and cardiology. Early results of the ORAMED project showed that the highest eye lens doses were measured during embolization procedures [37]. With increasing workload and complexity of the interventional cardiology procedures, the annual eye lens doses would be estimated to be relatively higher or even exceed the dose limits.

In summary, there is evidence of radiation-induced cataract risk at lower doses than previously realised and following protracted exposure. Although studies provide additional evidence for radiation causing damage to the eye, even at low doses, most studies in the literature do not allow assessment of the clinical impact of the radiation associated opacities [29]. Ionizing radiation exposure has been identified to link to vision-impairing cataracts in the A-bomb survivors in a recent study [39], although further research is needed to focus on interventional cardiologists regarding radiation exposure and development of cataracts. 

### 2.2. Radiation-Related Cardiovascular Diseases

Increased risk of cardiovascular diseases associated with ionizing radiation has received recent attention. Several studies have demonstrated the effects of ionizing radiation on hematologic parameters and immunologic function [40]; however, the question of whether radiation affects other physiologic phenomena, including arterial blood pressure, is still under debate despite continuous research efforts [41–43]. The key elements in radiation damage to vessels (microvessels in particular) are the endothelial cells [44]; however, much remains to be known about the intimate mechanisms of relationship between ionizing radiation and the endothelium damage. Experimental and human observations suggest that the endothelial cells are the most radioresponsive cells in the mesenchyma [45]. The initial response appears to be endothelial cell damage, leading to monocyte adhesion and transmigration into the subendothelial space. In the presence of elevated cholesterol levels, these invading monocytes transform into activated macrophages, which contribute to the formation of fatty streaks in the intima, resulting in the cascade of pathogenic changes that lead to radiation related heart disease [46–49]. Russo et al. analysed the response to chronic low-dose radiation by comparing 10 healthy interventional cardiologists with 10 unexposed controls by measuring hematological changes of redox state in lymphocytes [50]. The findings of their study demonstrate the association between low dose radiation and an altered redox balance which is manifested by an increase in hydrogen peroxide and adaptive cellular responses, although clinical meaning remains to be understood.

Early studies suggest that low-dose radiation can make human lymphocytes less susceptible to the genetic damage manifested as chromatic breakage induced by a subsequent high dose of X-rays [51, 52]. Later mortality analysis of atomic bomb survivors shows that radiation exposure increases cardiovascular disease mortality, suggesting that ionizing radiation accelerates blood vessel degeneration [53]. Increased mortality risk was reported for heart disease, stroke, and respiratory diseases in the Life Span Study of atomic bomb survivors, with an excess relative risk for death from heart disease of 0.14 per sievert [54]. Dose above 0.5 Gy was found to be associated with an elevated risk of both stroke and heart disease [55].

Epidemiological data on low dose radiation-induced damage to cardiovascular system are scare and conflicting: an increased cardiovascular disease risk was reported from studies of early radiologists in USA [56] but not from radiologists in the UK [57]. Studies of radiologists and radiologic technologists in the USA [58], Canada [59], Japan [60], Denmark [61], and China [62] lacked individual doses. Hauptmann et al. reported excess mortality from cardiovascular system based on their data on the US radiologic technologists [58], while other studies have not provided detailed analyses of cardiovascular disease. Their results showed that, for deaths from ischemic heart disease based on an analysis of 633 radiologic technologists, the relative risks were 0.98, 1.00, and 1.22 during 1950–1959, 1940–1949, or before 1940, compared with 1960 or later.

Although mortality from cardiovascular diseases increased with radiation dose among atomic bomb survivors, other epidemiological data investigating the association between low doses of ionizing radiation and circulatory diseases have not provided clear evidence of such a relationship. Yamada et al. in their cross-sectional analysis of atomic bomb survivors found that aortic calcification increases with radiation dose, thus, suggesting linkage between low-dose radiation dose and atherosclerotic cardiovascular changes. Prevalence of mild aortic arch calcification was found in 26.2% for men and 31.9% for women. There is a significant correlation between aortic calcification and radiation dose with dose more than 0.5 Gy resulting in significantly higher percentage of severe calcification when compared to the dose value of less than 0.5 Gy [63]. This is consistent with the dose limits of 0.5 Gy provided by ICRP [14]. Thus, medical professionals should be aware that the absorbed dose threshold for cardiovascular disease might be as low as 0.5 Gy to the heart.

A longitudinal study of the relationship atomic bomb exposure and cardiovascular disease in the Adult Health Study (AHS) has shown dose related increases in the incidence of stroke and myocardial infarction and in the incidence or prevalence of hypertension, elevated cholesterol levels in the exposed subjects [64–67]. The findings of the epidemiological study of cardiovascular disease have been further confirmed by a recently published study conducted by Shimizu et al. with more than 50 years of followup of 86611 atomic bomb survivors [68]. The study provides the strongest evidence so far that radiation dose may increase the prevalence of stroke and heart disease at moderate dose levels (mainly 0.5–2 Gy), although robust confirmatory evidence from other studies is needed.

In summary, data on the association between chronic low dose radiation and cardiovascular diseases are currently limited. Epidemiological studies are needed to help clarify the possible mechanisms between radiation exposure and its effect on the microcardiovascular damage.

## 3. Radiation-Induced Risks to Patients

Interventional cardiology procedures such as coronary angiography, percutaneous transluminal coronary angioplasty (PTCA), radiofrequency ablation, electrophysiological study, and left ventriculography contribute a significant proportion of radiation dose to patients due to the long fluoroscopy times and high-quality images required. Radiation doses can vary substantially across the same cardiac angiographic and interventional procedures, which is often a result of varying complexities of examination or patient size but can be a consequence of technological or procedural preference.

Patient dosimetry methods and quantities currently used in interventional cardiology can be divided into three categories [69]: (1) dosimetry for stochastic risk evaluation, which is associated with the risk of cancer induction; (2) dosimetry for quality assurance, which addresses evaluation of the optimization level of interventional cardiology procedures in comparison with performance of equipment and operator skill or comparison of the practice among different clinical centres; (3) dosimetry for deterministic effects of radiation exposure, which is related to the risk of deterministic injuries occurring. Dose quantities such as DAP (dose area product), fluoroscopy time, cine time, and number of cine images are useful indicators for evaluation of optimization level of interventional procedures [69, 70]. Effective dose is the most commonly used indicator in the assessment of diagnostic practice as it allows for estimation of the health risk due to stochastic effects of radiation.

### 3.1. Patient Dose

The radiation exposure to patients is determined by many factors, such as the X-ray equipment performance, the protocol used (e.g., frequency of cine frames, tube angulation, and the level of image quality), the operator skill and experience, the patient size, the interventional approach (e.g., femoral or radial technique), and several parameters related to the complexity and the nature of the intervention [71–74]. The Council Directive of the European Community 97/43 Euratom (MED) deals with the health protection of patients against risks of ionizing radiation associated with medical procedures and focuses on special procedures including interventional radiology [75]. Dose reference levels developed at the European level can be recommended in interventional cardiology examinations.

Delichas et al. found that the radiation dose to the patient is influenced by the individual characteristics of each interventional procedure [76]. The maximal annual dose values received by 9 cardiologists during 144 cardiac procedures in two hospitals in their study were found to be 1.9 and 2.8 mSv, which are much higher than the reference levels defined by the European DIMOND approach [77]. Tsapaki et al. reported differences of radiation exposure to patients between cardiologists with various levels of experience. Their results showed that the mean DAP values for coronary angiography and PTCA were 34.3 Gy × cm^2^ and 55.3 Gy × cm^2^ for cardiologists with more than 10 years of experience [78], while for cardiologists with less than 5 years of experience, the corresponding DAP values were 48.8 Gy × cm^2^ and 89.2 Gy × cm^2^, respectively. Bernardi et al. in their survey showed the correlation between radiation dose and types of interventional cardiology procedures with mean DAP being 65.8 Gy × cm^2^, 93 Gy × cm^2^, and 116.7 Gy × cm^2^, corresponding to the simple, medium, and complex procedure groups [79]. These reports emphasize the importance of minimizing radiation dose to patients during interventional cardiology procedures by taking into account the operator's experience and complexity of the procedures.

In summary, patient radiation dose varies widely not only among different interventional cardiology procedures but also among published studies. Discrepancies of the available results in the literature are patient-, procedure-, cardiologist-, and fluoroscopic equipment-related. Interventional cardiology procedures can expose patients to high radiation doses, thus, efforts to minimize patient exposure should always be undertaken.

### 3.2. Radiation-Induced Skin Injury

Skin is the organ at greatest risk during complex interventional procedures. Skin changes such as erythema, ulcers, telangiectasia, and dermal atrophy are well-known deterministic effects of ionizing radiation [80–82]. Although commonly referred to as skin injuries, severe radiation injuries can extend into the subcutaneous fat and muscle [83]. Patients may face years of associated pain, multiple surgical procedures, and permanent disfigurement [17, 84]. An early response (early transient ischemia) is noticed a few hours after doses of >2 Gy, when the exposed area is relatively large [14]. An actual skin dose in the 5–10 Gy range will always produce a noticeable injury with doses above 15 Gy leading to tissue being destroyed to a depth of a few centimetres and dermal necrosis [14, 85]. To minimize this risk, evaluation and assessment of maximum skin dose in interventional cardiology procedures are of paramount importance and should be recommended in the daily practice, although it is very difficult to undertake [86, 87].

Case reports describing deterministic radiation injuries on patient skin are increasing in the literature, and the potential for deterministic effects in some instances may be of more concern than stochastic long-term risk. Padovani and colleagues reported that the frequency of skin injuries in patients undergoing interventional cardiac procedures was less than 0.03% [70]. Kato et al. in their recent study observed 1.5% of radiation skin injury in 400 consecutive interventional cardiac procedures [88]. The higher rate of radiation injury in Kato's study may be due to the inclusion of many complicated interventional procedures, which result in high radiation doses exceeding the safe threshold level for skin. Radiation injuries are often misdiagnosed due to the late occurrence of their signs and symptoms, usually weeks after interventional procedures. Major injuries continue to progress for many months after the procedure. In most cases skin injuries have been reported in relationship with not optimised or improper use of radiological equipment due to the lack of knowledge of interventional cardiologists of radiological image formation, radiology technology, and radiation protection rules. It is a legal requirement to report significant radiological incidents and accidents that during or as a direct result of using ionizing radiation for a medical procedure. However, in practice, such reporting system is hardly implemented in many countries.

The IAEA has set up its own international reporting system called SAFety in RADiological procedures (SAFRAD) which is anonymous, so that it can be used for dose monitoring and reporting [89]. The reporting system includes patients who are exposed to defined trigger levels or events in fluoroscopically guided diagnostic and interventional procedures in an international database. The Safety in Radiation Oncology (SAFRON) is in the process of being developed by the IAEA to compile reports of medical radiation “incidents” that put patients at risk [90], and the IAEA smart card project on SmartRad Track makes tracking patient exposure a reality [91].

Development of online methods, based on calculation of skin dose distribution on the patient's skin, could be useful to alert the cardiologists when the regional skin dose exceeds the threshold for deterministic injuries [92]. Balter and Moses introduced a dose managing program in interventional cardiology [85]. This program uses a significant dose (reference point dose of 5 Gy for coronary procedures) as an action trigger for additional documentation and followup, which is designed to set low enough dose value to minimize the probability of missing a clinical deterministic injury. Similarly, Faulkner et al. proposed trigger levels for different interventional cardiology procedures based on different field sizes (DAP trigger level ranges from 20 to 400 Gy × cm^2^ corresponding to field size from 10 to 200 cm^2^), which may be used to identify patients at the risk of deterministic injuries [93].

In summary, radiation injury to human skin occurs at actual skin doses as low as a few gray. The dose-response relationships for both early and late radiation-induced damage to the skin are significantly influenced by the exposure rate. Increasing the dose above the injury threshold increases the degree of injury and prolongs the healing process.

## 4. Strategies to Reduce Radiation Risks to Interventional Cardiologists and Patients

Radiation exposure of interventional cardiologists and patients is currently a major concern. The National Council on Radiation Protection and Measurements recommends intraprocedure announcements of air-kerma to occur at 1000 mGy increments starting at 3000 mGy and recommends that specific postprocedure management practices are implemented following procedures with considerable radiation dose levels >5000 mGy [94]. Cardiac patients are increasingly exposed to cumulative diagnostic and therapeutic techniques of cardiac imaging using ionizing radiation, such as coronary angiography (average effective dose 5–10 mSv), PTCA (7–20 mSv), and nuclear cardiology (6–15 mSv) [95].

Patient and staff dosimetry on paediatric interventional cardiology procedures is another issue as cardiologists generally need to stay closer to the patient in comparison with adult procedures [96]. A prominent feature in paediatric fluoroscopy and intervention is the large size of the image intensifiers relative to the size of the neonate, infant, or child. The image intensifier will completely cover the patient and therefore has the potential to increase radiation exposure if collimation is not used [97]. Reports involving the evaluation of paediatric cardiology procedures are limited. Ubeda et al. in their multicentre study indicated the large variability in paediatric interventional cardiology protocols and imaging parameters (including kV and mA ranges for fluoroscopy and cine modes), with measured scatter dose ranging from 0.8 to 12 mSv h^−1^ at the eye position during fluoroscopy and cine modes if no protective tools are used [96].

Several aspects of radiation safety in the interventional cardiology have been proposed with effective dose reduction outcomes having been achieved. These include strategies of dose monitoring during the procedure, wearing protective devices, applying dose-reduction techniques, and implementing training and education programmes.

### 4.1. Dose Monitoring

It is necessary for interventional cardiologists to wear dosimeters on a regular basis. Occupational dosimetry is critical for the personal safety of interventional cardiologists. The ICRP and ACC recommend the use of two personal dosimeters, with one worn outside the apron at the shoulder or neck and the other worn under the apron at the waist [6, 98]. In addition, special dosimeters could be used for special practices (e.g., a ring dosimeter for biliary drainage/stent procedure which involves delivery of high doses to the hands) to monitor doses to the skin, hands, feet, and the lens of eyes.

Martin conducted a review of the dose data from studies of radiology performed over the last 20 years which involved X-ray procedures by radiology, cardiology, and other medical staff [99]. The doses in the studies reviewed vary by factors of 60–100 for similar procedures in different centres. As the number of interventional procedures is gradually increasing and the potential for medical staff to receive high doses, it is important to ensure that doses received by interventional cardiologists are monitored to check whether the protection devices are deployed effectively.

### 4.2. Use Protective Shielding and Wear Protective Devices

Equipment-mounted shielding includes protective drapes suspended from the table and from the ceiling. Ceiling-suspected shields can provide substantial dose reduction, especially to the unprotected areas such as head and neck. Under-table lead drapes reduce lower extremity dose substantially and should be used whenever possible [100]. They should always be employed, as they have been reported to significantly reduce operator dose [101].

**Figure 1:**
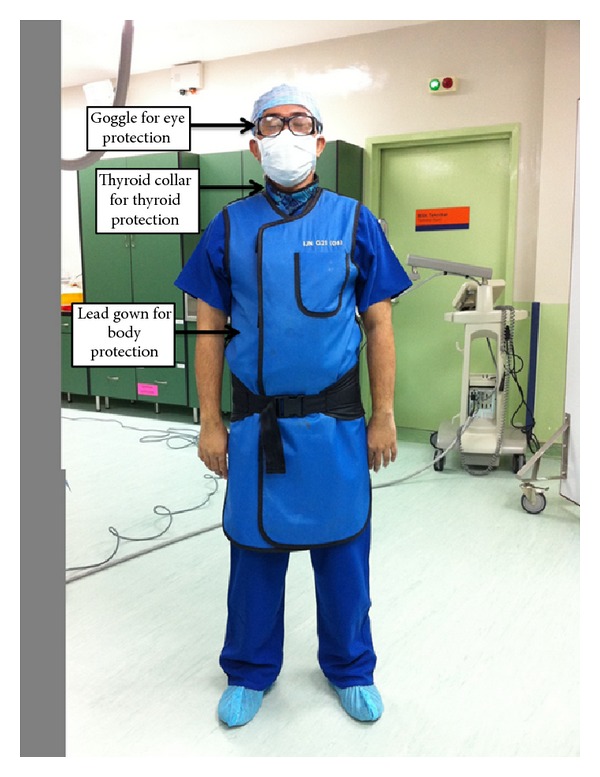
Wearing protective devices during interventional cardiology procedures.

It has been reported that interventional cardiologists received an average annual effective dose of 46.2 mSv without wearing protective devices [102]. The dose can be reduced to 3.5 mSv per year using a lead apron and to 1.7 mSv per year using both a lead apron and a thyroid shield (Figure 1) [103]. Whereas the effective dose is routinely assessed by dosimetry, less attention is given to the local scatter radiation doses to unprotected parts of the body, especially the head and hands as cardiologists are also exposed to scatter radiation. Thus, wearing protective devices should be highly recommended during interventional cardiology procedures.

**Figure 2:**
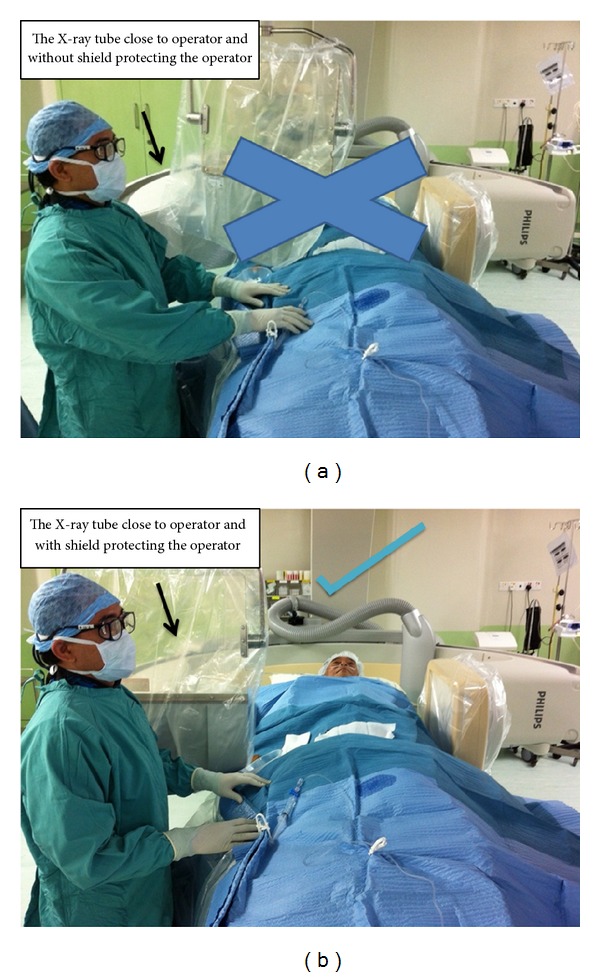
(a) The lateral projection is not recommended when the lead shield is not protecting the operator. (b) The lateral projection is recommended when the lead shield is protecting the operator.

In cardiac intervention, radiation shields are widely used to reduce scatter radiation; however, the use of protective shields plays an essential role in optimizing protection during the interventional cardiology procedures (Figure 2). Fetterly et al. in their study reported that radiation shields must be thoughtfully placed and actively managed both before and during the procedure to be effective in providing substantial protection from radiation during interventional cardiology procedures [104]. Best practice guidelines for shield use are provided in their study with regard to upper body protection, lower body shields, or different approaches relating to different interventional procedures.

A leaded glass or plastic screen placed between the patient and the operator protects the operator's eyes, head, and neck. Leaded eye glasses with protective side shields provide more protection than eye glasses without these features [105]. Properly placed shields have been shown to reduce operator eye dose significantly [106, 107]. These screens add no weight to the operator, eliminating the ergonomic consequences of the protective equipment; thus they can effectively replace both leaded eyewear [7]. Maeder et al. reported the effectiveness of reduction of scatter radiation to the eyes with use of a transparent lead glass screen, but minimal effects on the dose to the hands [106]. Therefore, additional efforts are required to reduce dose levels to both patients and operators.

### 4.3. Education and Training Programmes

It is important for the medical profession and other healthcare professionals to be aware of the hazards from radiation in order to avoid the unnecessary risks to the population as a whole. Lack of knowledge may result in more ionizing radiation imaging examinations being requested when other nonradiation tests could be performed or when different lower-dose imaging tests could be performed. This is particularly important for interventional cardiology procedures as they deliver high doses to medical staff and patients. As such, the need for education and training in radiological protection is more compelling.

The European Commission and ICRP have addressed the importance of training in radiological protection, publishing guidelines with specific recommendations for accreditation of training programmes for interventional procedures [108, 109]. ICRP Publication 113 recommends that training in radiological protection is included in the quality assurance programme, with special attention to training given to fellows and residents [109]. The guideline provided by European Commission suggests specific learning objectives and 20–30 hours of training for interventional cardiologists. Much effort has been made over the last decade to produce training materials to help improvement of radiation protection in interventional cardiology procedures, with successful outcomes having been achieved [85, 95, 110]. The educational programme has been shown to be effective at improving compliance with the radiation badge monitoring programme [111]. Cardiology scientific societies should promote training activities in radiation protection to maintain a high level of radiation safety in the practice of interventional cardiology. The Guidance Document developed as work package 3 of the MEDRAPET project is expected to be used as the basis for curricula of radiation protection courses [112].

There is an urgent need to implement and propagate widely the training programs in interventional cardiology such as the IAEA initiated radiation cataract study called Retrospective Evaluation of Lens Injuries and Dose (RELID), which is organized in collaboration with professional societies of cardiologists in many countries [113].

### 4.4. Dose Reduction Techniques

Operational measures play an important role in improving radiation protection and dose reduction to both medical staff and patients. Decreasing patient dose will result in a proportional decrease in scatter dose to the operator; therefore, techniques that reduce patient dose will generally reduce occupational dose. A practical advice to reduce or minimize the occupational radiation dose has been recently proposed by the Cardiovascular and Interventional Society of Europe (CIRSE) [101].  Table 1 summarises various dose-reduction techniques and the corresponding functions.

One of the most important radiation protection measures is to increase patient's distance from the radiation source. Working at 80 cm from the isocenter instead of 40 cm can decrease scattered dose to approximately 25% of the original dose [114]. Variable dose values associated with radial versus femoral artery access have been reported in the literature [115, 116]. Fetterly et al. demonstrated that patient dose was reduced simultaneously with increased utilization of radial access [104]. Other technical alterations provide the potential for systematic dose reduction, including a change from continuous fluoroscopy to pulsed fluoroscopy modes; reduction in fluoroscopy/acquisition frame rate from 30 frames/s to 7.5/15 frames/s; collimation of the radiation field to decrease the level of scattered dose; improved X-ray image detection and display systems; and increased use of metallic X-ray beam spectral filters for both fluoroscopy and acquisition imaging [104, 114]. Improved image processing within the fluoroscopic unit can compensate to a greater extent for the reduced image quality due to decreased exposure levels.

Diagnostic reference levels (DRLs) are radiation dose values for specific examinations that should not be consistently exceeded when good practice is in place and if regularly exceeded remedial action should be sought. DRLs are used to help avoid radiation dose to the patient that does not contribute to the medical imaging task. They are intended to provide guidance on what is achievable with current good practice rather than optimum performance and help to identify unusually high radiation doses or exposure levels [7]. These are legal requirements in a number of jurisdictions [117, 118] and have been shown to be very effective in reducing dose and dose variations for a variety of investigations since their introduction two decades ago.

## 5. Summary and Conclusion

In recent years, intensive efforts have been initiated to reduce the radiation dose associated with interventional cardiology. It has been become a routine practice for publications addressing cardiac intervention to report radiation doses. There is increasing concern about the potential deleterious effects from radiation arising from intervention cardiology due to two reasons: first, cardiac procedure volumes have grown tremendously. Second, the radiation doses received by interventional cardiologists and patients can vary by more than an order of magnitude for the same type of procedure. Increased workload, complexity of the interventional procedures, and acute patient conditions contribute significantly to the amount of radiation exposure to both patients and medical staff.

Noncancer risks of radiation in interventional cardiology that have been discussed in different scenarios emphasize the importance of reducing radiation dose to patients and medical staff. This can be achieved through implementing necessary strategies such as continual improvements in protocols and equipment, implementation of guidelines proposed by professional bodies into daily practice and attending training programmes to ensure best practice. Epidemiological studies involving a large cohort of individuals exposed to ionizing radiation will provide us with a full picture as to the true effects of radiation exposure from interventional cardiology. A final general recommendation is that being aware of the radiological protection of your patient will also be improving your own occupational protection.

## Figures and Tables

**Table 1:** Dose reduction techniques that are commonly used in interventional cardiology procedures.

Techniques used in interventional cardiology	Corresponding functions
Minimize use of fluoroscopy time and use low fluoroscopy mode	Reduce staff and patient dose
Number of fluorographic images	Reduce staff and patient dose
Image-chain geometry	Reduce patient dose
Collimation of the radiation field	Decrease the level of scatter dose
Medical staff position in a low-scatter area	Reduce staff dose
Wear protective shielding	Reduce radiation dose to eye lens and other organs
Fluoroscopic imaging equipment comply with International Electrotechnical Commission [119]	Dose-reduction technology is incorporated into the imaging systems
Obtain appropriate training provided by professional bodies	Increase awareness of radiation protection and dose reduction
Wear personal dosimeter	Know and monitor your own dose
Diagnostic reference levels	Monitor clinical practice and radiation dose
